# Clinical Effectiveness of Various Surgical Procedures Addressing Long Head of Biceps Pathology: Protocol for a Systematic Review and Meta-Analysis

**DOI:** 10.29337/ijsp.139

**Published:** 2021-04-13

**Authors:** Alexander W. Hartland, Raisa Islam, Kar H. Teoh, Mustafa S. Rashid

**Affiliations:** 1 Broomfield hospital, Court Road, Broomfield, Chelmsford, CM1 7ET, UK; 2 Trauma and Orthopaedics, Princess Alexandra Hospital, Hamstel Road, Harlow, Essex, CM20 1QX, UK; 3 Nuffield Department of Orthopaedics, Rheumatology, and Musculoskeletal Sciences, Windmill Road, Oxford, OX3 7LD, UK; 4 Botnar Research Centre, Windmill Road, Oxford, OX3 7LD, UK

**Keywords:** Biceps, Biceps brachii, Tenodesis, Tenotomy, Transfer, Long head

## Abstract

**Introduction:**

The long head of biceps tendon is a common source of anterior shoulder pain and impaired function. Multiple surgical procedures are available as treatment options, but the optimal procedure is not known. The aim of this systematic review and meta-analysis is to review the literature to assess the clinical effectiveness of various surgical procedures to treat pain arising from the long head of biceps.

**Methods:**

The study protocol was designed and registered prospectively on PROSPERO (International prospective register for systematic reviews). Electronic databases used for the literature search will include MEDLINE, EMBASE, PsycINFO, and The Cochrane Library. Randomised controlled trials (RCTs) evaluating surgical procedures on the long head of biceps will be included. Our primary outcome is any functional patient-reported outcome measure related to the shoulder. Secondary outcomes will include the rate of ‘Popeye’ deformity, the rate of biceps cramping pain, the rate of complications, objective measurements of strength testing such as dynamometer, and other patient-reported outcome measures not specific to the shoulder such as the Visual-Analog Scale (VAS) for pain. Methodological quality of included studies will be assessed using The Cochrane Risk of Bias Tool 2.0 and the Jadad score. Inconsistency and bias across included studies will be assessed statistically. Comparable outcome data will be pooled and analysed quantitatively or qualitatively as appropriate.

**Ethics and dissemination:**

No ethical clearances required for this study. We plan to publish this systematic review and meta-analysis in a peer-reviewed journal. It will also be presented at various national and international conferences.

**Highlights:**

- Evaluating the clinical effectiveness of surgical procedures for long head of biceps pathology.
- Randomised controlled trials.
- Biceps tenodesis and biceps tenotomy.
- Systematic review compliant with the PRISMA guideline.

## 1. Introduction

The long head of biceps tendon is a common source of anterior shoulder pain [1,2,3] and impaired function [4]. Pathology involving the long head of biceps tendon can be primary or secondary in nature. Primary pathology involves long head of biceps tendinopathy including fraying and tears. Secondary pathology occurs in association with other diagnoses, frequently rotator cuff tears [3,5]. Conservative management of long head of biceps tendinopathy can include rest, ice compress, oral non-steroidal anti-inflammatory drugs, corticosteroid intra-articular injection, and physical therapy [5]. When surgical management is required, the optimal surgical procedure to provide optimal clinical benefit remains unclear. Various surgical procedures have been described. A popular option is a biceps tenotomy. This is a technically simple and well tolerated procedure with minimal post-operative rehabilitation. Some patients report adverse effects including ‘Popeye’ deformity, cramps, and fatigue [6,7,8,9]. Biceps tenodesis is a common alternative, which may be favoured in some patients and allows a closer restoration of normal anatomy [9,10]. This procedure is technically more difficult and requires a longer post-operative rehabilitation period [7,9,11]. A wide variety of techniques have been described for biceps tenodesis [12,13,14,15]. These can be categorised by location (intra-articular, extra-articular but within the bicipital grove, and sub-pectoral). The method of tenodesis can also be further categorised into suture anchors, suspensory buttons, and screw fixation. O’Brien reported a technique for arthroscopic long head of biceps tendon transfer and soft tissue tenodesis to the conjoint tendon [16].

Most techniques have been reported in observational studies and have shown good to excellent outcomes in the vast majority. Few comparative studies have been reported, directly comparing two or more techniques. Despite these, there remains some dissonance amongst the published literature. The purpose of this meta-analysis and systematic review is to assess the clinical effectiveness of various surgical procedures to treat pain arising from the long head of biceps within the shoulder.

## 2. Methods

This study protocol was designed and prospectively registered on the PROSPERO (International prospective register for systematic reviews) database (Ref: CRD42020198658). The protocol is reported according to the Preferred Reporting Items for Systematic reviews and Meta-Analyses Protocol (PRISMA-P) [17,18].

### 2.1 Eligibility criteria

#### 2.1.1 Study design

Randomised controlled trials will be included. All other trial designs will be excluded.

#### 2.1.2 Participants

We will include studies with human patients of any age undergoing any type of surgery to the long head of biceps. This may include arthroscopic and open techniques.

#### 2.1.3 Intervention and comparators

The intervention of interest is a surgical procedure of biceps tenodesis. The comparators will be alternative surgical procedures for biceps pathology including, biceps tenotomy and tendon transfer.

#### 2.1.4 Outcomes

The primary outcome of interest will be any functional patient-reported outcome measures related to the shoulder. This may include The Oxford Shoulder Score (OSS), The Constant-Murley Score (CMS), and The American Shoulder and Elbow Surgeons Shoulder Score (ASES).

Secondary outcomes will include the rate of ‘Popeye’ deformity, the rate of biceps cramping pain, the rate of complications such as humeral fractures, objective measurements of strength testing, and other patient-reported outcome measures not specific to the shoulder such as the Visual-Analog Scale (VAS) for pain.

#### 2.1.5 Timing

No restrictions on the timing of the study. Where multiple studies report on the same patient cohort exist, the study with the longest time to follow-up will be included.

#### 2.1.6 Setting

No restrictions on the setting of the study.

#### 2.1.7 Language

No restrictions on the language of the study. Any studies that require translation into English will be included in the appendix.

### 2.2 Information sources

The following bibliographic databases were searched; MEDLINE, EMBASE, PsycINFO and The Cochrane Library.

#### 2.2.1 Search strategy

No restrictions were placed on the date of publication. In order to increase sensitivity and heighten precision randomised controlled trial filters, provided by The Cochrane group, were used for each database in the search strategy. The utilised search terms are included in the appendix. We manually searched references from published systematic reviews investigating the same or similar topic for relevant included studies. On searching the PROSPERO database, no ongoing or recently completed systematic reviews on this exact topic were found.

### 2.3 Study records

#### 2.3.1 Data management

All literature search results will be combined and collected in Endnote X9 (Clarivate Analytics) with duplicate articles being removed. Two independent reviewers will screen titles and abstracts of returned search results, with consensus sought prior to full text review. Subsequent full text review of articles meeting all eligibility criteria will determine the final inclusion.

#### 2.3.2 Data collection process

Data extraction will involve two independent reviewers. A standardised proforma will be used by one reviewer to extract the required data. A second reviewer will then check the extracted data for any inaccuracies. Any differences found during the data extraction process will be resolved by discussion and the involvement of a third reviewer as needed. Authors of individual studies will be attempted to be contacted regarding any missing data or any desired additional information. Microsoft Excel will be used for data capture and Review Manager (RevMan version 5.3) used as a software tool for data management.

#### 2.3.3 Data items

Extracted data items will include study design, patient cohort, study characteristics, surgical intervention, comparator surgical intervention, primary outcome measure data, and any secondary outcome measure data. Mean and standard deviations will be extracted for all outcome measures. Data on adverse events will be extracted.

### 2.4 Outcomes and prioritisation

#### 2.4.1 Primary outcome

The primary outcome of interest will be functional patient-reported outcome measures related to the shoulder. This may include The Oxford Shoulder Score (OSS), The Constant-Murley Score (CMS), and The American Shoulder and Elbow Surgeons Shoulder Score (ASES).

#### 2.4.2 Secondary outcomes

Secondary outcomes examined will include the rate of ‘Popeye’ deformity, the rate of biceps cramping pain, the rate of complications such as humeral fractures, objective measurements of strength testing, and other patient-reported outcome measures not specific to the shoulder such as the Visual-Analog Scale (VAS) for pain.

### 2.5 Risk of bias of individual studies

To assess for potential bias of individual studies, the Cochrane collaboration Risk of Bias tool 2.0 will be used [19]. Within this tool, there are 5 domains of bias, with each domain being assigned a level of risk of bias (high risk, low risk, or some concerns). Interpretation of the risk of bias for each domain will be guided by pre-set signalling questions. The tool subsequently generates an overall risk of bias for each study. As a supplementary method for assessing bias, each study will also be assessed using the Jadad scale [20]. The Jadad scale ranges from 0 to a maximum of 5 points. 2 points can be given for randomisation – 1 point for stating the study is randomised and a further point if the method of randomisation is appropriate. 2 points can be given for blinding – 1 point for stating the use of blinding within the study and a further point if the method of blinding is appropriate. An additional point is given if all patients involved in the trial have been accounted for.

### 2.6 Data synthesis

#### 2.6.1 Quantitative synthesis

Data will be synthesised quantitatively, in the form of a forest plot, if the outcomes recorded within individual studies are comparable. We will assess for heterogeneity between studies. Heterogeneity will be quantified using the using chi-square test for heterogeneity and the I^2^ statistic. Due to expected heterogeneity between studies, a random effects model is likely to be used for most analyses. Data from continuous variables will be summarised using standardised mean difference and inverse variance statistical analysis. Any dichotomous data presented will be measured for effect using odds ratios.

#### 2.6.2 Qualitative synthesis

Data will only be reported descriptively when outcome measures from individual studies are not comparable, heterogeneity is too high, or the rate of incidence of the event is too low for pooled statistical analysis.

#### 2.6.3 A priori subgroup analyses

We expect to be able to perform multiple subgroup analyses based on our inclusion criteria. This may include isolated biceps pathology versus biceps pathology with associated rotator cuff repair, younger patients versus older participants (possible age range for treatment effect), type of fixation used during biceps tenodesis (screws, anchors, or buttons), as well as, the tenodesis location (intra-articular, subpectoral, or extra-articular but within the bicipital groove).

#### 2.6.4 Meta-bias

Meta-biases will be assessed for by assessing publication bias, with use of a funnel plot of included studies investigating our primary outcome. Reviewing available trial protocols or registrations to compare pre-defined outcomes with those ultimately analysed and reported, will also assess for selective reporting within studies. The risk of bias within each individual study will be assessed for as previously described. Statistical analysis of heterogeneity, as a measure of inconsistency, will be used to assess bias across studies.

#### 2.6.5 Confidence in cumulative estimate

The strength of the body of evidence provided will be assessed using the Grading of Recommendations Assessment, Development and Evaluation (GRADE) approach [21,22,23]. Each outcome assessed will consequently be described as being of very low, low, moderate, or high certainty.

## Appendix

### Search terms for MEDLINE

1. Randomised controlled trial.pt.
2. Controlled clinical trial.pt.
3. Randomised.ab,ti.
4. Placebo.ab,ti.
5. Clinical trials.mp.
6. Randomly.ab,ti.
7. Trial.ti.
8. 1 OR 2 OR 3 OR 4 OR 5 OR 6 OR 7
9. Bicep.ab,ti.
10. Biceps.ab,ti.
11. Biceps brachii.ab,ti.
12. Bicipital.ab,ti.
13. 9 OR 10 OR 11 OR 12
14. Tenotomy.ab,ti.
15. Tenodesis.ab,ti.
16. Transfer.ab,ti.
17. Reattach.ab,ti.
18. Reattached.ab,ti.
19. Reattachment.ab,ti.
20. Surgical.ab,ti.
21. Surgery.ab,ti.
22. Operative.ab,ti.
23. Operation.ab,ti.
24. Procedure.ab,ti.
25. Fixation.ab,ti.
26. 14 OR 15 OR 16 OR 17 OR 18 OR 19 OR 20 OR 21 OR 22 OR 23 OR 24 OR 25
27. 8 AND 13 AND 26

## Registration

PROSPERO 2020 CRD42020198658

## References

[1] Corpus KT, et al. Long Head of Biceps Tendon Management: a Survey of the American Shoulder and Elbow Surgeons. HSS J. 2018; 14(1): 34–40. DOI: 10.1007/s11420-017-9575-3 29398992 PMC5786587

[2] Patel KV, et al. Biceps Tenotomy Versus Tenodesis. Clin Sports Med. 2016; 35(1): 93–111. DOI: 10.1016/j.csm.2015.08.008 26614471

[3] Wilk KE, Hooks TR. The Painful Long Head of the Biceps Brachii: Nonoperative Treatment Approaches. Clin Sports Med. 2016; 35(1): 75–92. DOI: 10.1016/j.csm.2015.08.012 26614470

[4] Ditsios K, et al. Long head of the biceps pathology combined with rotator cuff tears. Adv Orthop. 2012; 2012: 405472. DOI: 10.1155/2012/405472 23209915 PMC3507080

[5] Ahrens PMBP. The long head of biceps and associated tendinopathy. J Bone Joint Surg. 2007; 89-B: 1001–9. DOI: 10.1302/0301-620X.89B8.19278 17785735

[6] Meeks BD, et al. Patient Satisfaction After Biceps Tenotomy. Orthop J Sports Med. 2017; 5(5). DOI: 10.1177/2325967117707737 PMC5448732 28596975

[7] Friedman JL, et al. Biceps Tenotomy Versus Tenodesis in Active Patients Younger Than 55 Years: Is There a Difference in Strength and Outcomes? Orthop J Sports Med. 2015; 3(2). DOI: 10.1177/2325967115570848 PMC4555607 26535382

[8] Mirzayan R, et al. Risk Factors and Complications Following Arthroscopic Tenotomy of the Long Head of the Biceps Tendon. Orthop J Sports Med. 2020; 8(2). DOI: 10.1177/2325967120904361 PMC7052466 32166093

[9] Na Y, et al. A meta-analysis comparing tenotomy or tenodesis for lesions of the long head of the biceps tendon with concomitant reparable rotator cuff tears. J Orthop Surg Res. 2019; 14(1): 370. DOI: 10.1186/s13018-019-1429-x 31729995 PMC6858715

[10] Werner BC, Brockmeier SF, Gwathmey FW. Trends in long head biceps tenodesis. Am J Sports Med. 2015; 43(3): 570–8. DOI: 10.1177/0363546514560155 25497144

[11] Shang X, Chen J, Chen S. A meta-analysis comparing tenotomy and tenodesis for treating rotator cuff tears combined with long head of the biceps tendon lesions. PLoS One. 2017; 12(10): e0185788. DOI: 10.1371/journal.pone.0185788 29016616 PMC5633150

[12] Lo I, Burkhart S. Arthroscopic biceps tenodesis using a bioabsorbable interference screw. Arthroscopy. 2004; 20(1): 85–95. DOI: 10.1016/j.arthro.2003.11.017 14716285

[13] Javed S, Gheorghiu D, Walton M. Subpectoral biceps tenodesis using a novel anterior cortical button technique. Shoulder Elbow. 2018; 10(4): 292–295. DOI: 10.1177/1758573218778799 30214496 PMC6134529

[14] Beletsky A, et al. Arthroscopic Tenodesis of the Long Head Biceps Tendon Using a Double Lasso-Loop Suture Anchor Configuration. Arthrosc Tech. 2019; 8(10): e1137–e1143. DOI: 10.1016/j.eats.2019.05.028 31921587 PMC6948130

[15] Gifford A, et al. Mini-open Subpectoral Biceps Tenodesis Using All-Suture Anchor. Arthrosc Tech. 2020; 9(4): e445–e451. DOI: 10.1016/j.eats.2019.11.017 32368463 PMC7189024

[16] Verma NN, Drakos M, O’Brien SJ. Arthroscopic transfer of the long head biceps to the conjoint tendon. Arthroscopy. 2005; 21(6): 764. DOI: 10.1016/j.arthro.2005.03.032 15944642

[17] Moher D, et al. Preferred reporting items for systematic reviews and meta-analyses: the PRISMA statement. BMJ. 2009; 339: b2535. DOI: 10.1136/bmj.b2535 19622551 PMC2714657

[18] Shamseer L, et al. Preferred reporting items for systematic review and meta-analysis protocols (PRISMA-P) 2015: elaboration and explanation. BMJ. 2015; 350: g7647. DOI: 10.1136/bmj.g7647 25555855

[19] Sterne JAC, et al. RoB 2: a revised tool for assessing risk of bias in randomised trials. BMJ. 2019; 366: l4898. 31462531 10.1136/bmj.l4898

[20] Jadad ARM, Carroll RA, Jenkinson D, Reynolds C, Gavaghan DJM, McQuay DJHJ. Assessing the Quality of Reports of Randomized Clinical Trials: Is Blinding Necessary? Controlled Clin Trials. 1996; 17: 1–12. DOI: 10.1016/0197-2456(95)00134-4 8721797

[21] Balshem H, et al. GRADE guidelines: 3. Rating the quality of evidence. J Clin Epidemiol. 2011; 64(4): 401–6. DOI: 10.1016/j.jclinepi.2010.07.015 21208779

[22] Guyatt GHO, Vist AD, Kunz GE, Falck-Ytter R, Alonso-Coello Y, Schunemann PHJ. GRADE: an emerging consensus on rating quality of evidence and strength of recommendations. BMJ. 2008; 336: 924–926. DOI: 10.1136/bmj.39489.470347.AD 18436948 PMC2335261

[23] Langer G, et al. GRADE guidelines: 1. Introduction – GRADE evidence profiles and summary of findings tables. Z Evid Fortbild Qual Gesundhwes. 2012; 106(5): 357–68. DOI: 10.1016/j.zefq.2012.05.017 22818160

